# A Review on Ubiquitination of Neurotrophin Receptors: Facts and Perspectives

**DOI:** 10.3390/ijms18030630

**Published:** 2017-03-14

**Authors:** Julia Sánchez-Sánchez, Juan Carlos Arévalo

**Affiliations:** 1 Department of Cell Biology and Pathology, Institute of Neuroscience Castile & Leon, University of Salamanca, 37007 Salamanca, Spain; juliasanchez@usal.es; 2 Institute of Biomedical Research of Salamanca, 37007 Salamanca, Spain

**Keywords:** deubiquitination, neurotrophins, p75^NTR^, Trk receptors, ubiquitination

## Abstract

Ubiquitination is a reversible post-translational modification involved in a plethora of different physiological functions. Among the substrates that are ubiquitinated, neurotrophin receptors (TrkA, TrkB, TrkC, and p75^NTR^) have been studied recently. TrkA is the most studied receptor in terms of its ubiquitination, and different E3 ubiquitin ligases and deubiquitinases have been implicated in its ubiquitination, whereas not much is known about the other neurotrophin receptors aside from their ubiquitination. Additional studies are needed that focus on the ubiquitination of TrkB, TrkC, and p75^NTR^ in order to further understand the role of ubiquitination in their physiological and pathological functions. Here we review what is currently known regarding the ubiquitination of neurotrophin receptors and its physiological and pathological relevance.

## 1. Neurotrophins and Their Receptors

**Figure 1:**
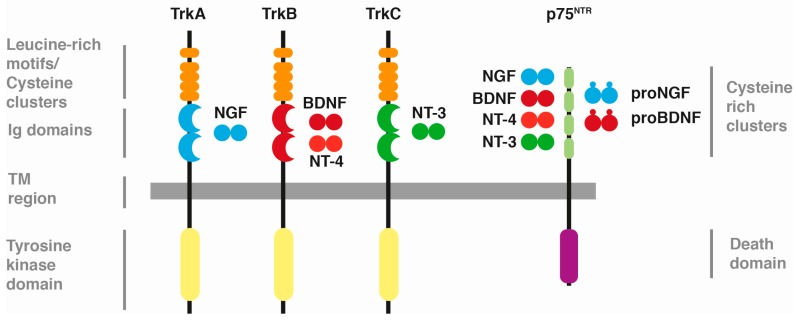
Neurotrophins and their receptors. (Adapted from Arévalo and Wu [14]). Scheme showing the common structure and ligands of Trk receptors, as well as in p75^NTR^. Trk: Tropomyosin Related Kinase; NGF: Neuronal Growth Factor; BDNF: Brain-Derived Neurotrophic Factor; NT: Neurotrophin; Ig: Immunoglobulin; TM region: Transmembrane region.

The mammalian nervous system requires well-established neuronal function that depends on the proper survival, differentiation, and axonal and dendritic growth of neurons, among other important cellular events. To achieve this, neurotrophin-mediated signalling is one of the main ways the neural network is acquired and maintained [1,2]. The neurotrophin family comprises four different members. The first of these growth factors to be described was the neuronal growth factor (NGF) [3], followed by the discovery of the brain-derived neurotrophic factor (BDNF) [4], neurotrophin-3 (NT-3) [5], and neurotrophin-4/5 (NT-4/5) [6,7]. The actions mediated by these molecules depend on their binding to two different subsets of transmembrane receptors. On one side, the pro-neurotrophins (immature form of these factors) and mature neurotrophins bind to the p75 neurotrophin receptor (p75^NTR^), and on the other, only mature neurotrophins bind to three different tropomyosin-related kinase (Trk) receptors in a specific manner. In this way, NGF binds to TrkA, BDNF and NT-4/5 to TrkB, and NT-3 to TrkC [2] (Figure 1). Following ligand binding, Trk receptors dimerise and promote the transphosphorylation of the cytoplasmic tyrosine-kinase domains and subsequent activation of pathways that regulate the functions of neurotrophins. While Trk-mediated signalling usually promotes neuronal survival and growth as well as synapse modulation, p75^NTR^ activation counteracts these processes. Nevertheless, p75^NTR^ can also act as a Trk co-receptor, allowing a stronger binding affinity of the neurotrophins to the Trk receptors [8]. Once the activation of Trk receptors takes place, they are internalised along with other phosphorylated proteins downstream of the Trk pathway, suggesting that the signalling remains after the receptor is removed from the cell surface [9,10]. Following the phosphorylation of their cytoplasmic domain after neurotrophin binding occurs, Trk receptors can be regulated via ubiquitination [11,12,13]. Through this recently described modulation of Trk receptors, events such as turnover and/or degradation take place. Here we review the current knowledge regarding the ubiquitination of neurotrophin receptors and its physiological implications in neurotrophin-mediated functions.

## 2. Ubiquitination

**Figure 2:**
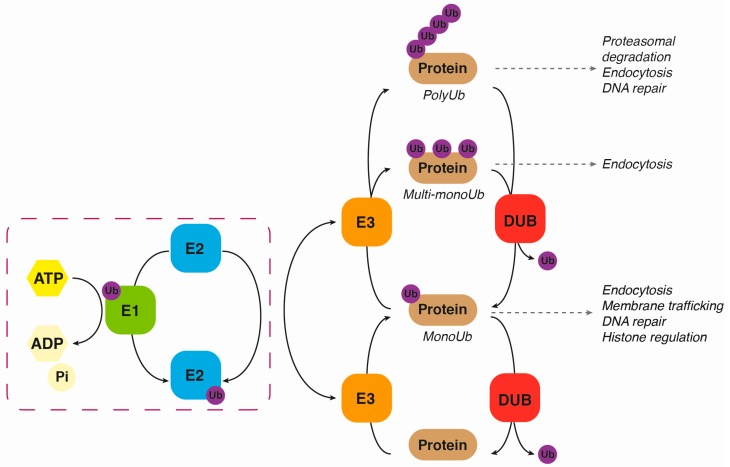
Ubiquitination/deubiquitination process. (Adapted from Fraile et al. [24]). Summary of the steps involving the ubiquitination and deubiquitination of proteins. DUB: Deubiquitinase; E1: ubiquitin activating enzyme; E2: ubiquitin conjugating enzyme; E3: ubiquitin ligase enzyme; Ub: ubiquitin.

Ubiquitination is a reversible post-translational modification in which a 76-amino-acid polypeptide called ubiquitin (Ub) is attached to proteins. Originally, it was described as a way to target misfolded or short-lived proteins for proteasomal degradation [15]. Nevertheless, it has been widely reported that this action could lead to other outcomes, acquiring a new and prominent role in protein signalling [16]. Regardless of whether the purpose of ubiquitination is one or the other, this process involves the covalent binding of Ub to a protein via three sequential enzymatic steps. The first step is ATP-dependent and comprises the activation of Ub by an Ub-activating enzyme (also called E1), in which a thiol-ester linkage is formed between the active cysteine residue of the E1 and the C-terminus of Ub. Afterwards, the activated Ub is transferred to the cysteinyl group of the Ub-conjugating enzyme (E2), which forms a complex that interacts with the third enzyme of this process—a Ub-ligase (E3). Finally, the ubiquitin is transferred either directly from the E2 via an E3 Ub-ligase or through previous binding to the E3 Ub-ligase [17]. After considering the two different mechanisms and the different domains they contain, we can divide the E3 Ub-ligases into three different families: the RING (really interesting new gene)-finger family, the HECT (homologous to E6AP carboxy terminus) family and the U-box (UFD2 homology) family [18,19,20]. The HECT members collect Ub from the E2 and then transfer Ub to the substrate, while RING and U-box Ub-ligases act as a scaffold that allows the transfer of Ub from the E2 to the protein (Figure 2). In addition to just one Ub molecule attaching to the substrate (monoubiquitination), a single Ub molecule can also become attached to multiple lysine (K) residues on the same substrate, leading to a multi-monoubiquitination. Moreover, Ub molecules can attach to each other end-to-end on the same lysine residue on a substrate protein, forming a polyubiquitin linear chain. It also happens that some Ub-ligases can form branched ubiquitin chains that seem to enhance the ubiquitin signal [21]. This can result in the formation of different types of ubiquinated proteins, depending on the lysine where Ub is attached (K6, K11, K27, K29, K33, K48, K63). Finally, it is possible to generate another variant that consists of a head-to-tail linear polyubiquitin chain in which the C-terminal Gly of one ubiquitin monomer is conjugated to the N-terminal Met of the next ubiquitin monomer [22]. Therefore, different types of protein ubiquitination can lead to different protein fates, such as degradation, signal transduction, intracellular trafficking of transmembrane proteins, among others [23]. For example, when the polyubiquitination of K48 takes place, the protein ends up being degraded by the proteasome, whereas the K63-linked polyubiquitination—which does not prime the substrate for proteasome-dependent degradation—regulates cell functions such as signal transduction or intracellular trafficking. As previously mentioned, different mechanisms have recently been described where neurotrophin receptors can be modulated through the ubiquitination process. We will thoroughly describe these findings in the sections that follow.

## 3. Deubiquitination

Ubiquitination is a highly specific process which can be reversed by specific proteases. In such a way, deubiquitinases (DUBs; the enzymes that catalyse the removal of ubiquitin) are responsible for the modulation of proteasome and lysosome degradation, polyubiquitin-chain editing, and the removal of the Ub attached to the proteins, participating in its maturation, recycling, and editing [25,26]. Around one hundred DUBs have been described within the human genome, which can be classified in two groups depending on their proteolytic action. In one group, there are the cysteine proteases that can be subdivided into four different families according to their protease domain: the ubiquitin C-terminal hydrolases (UCH), the ubiquitin-specific proteases (USP), the ovarian tumour proteases (OTU) and the Machado–Joseph disease proteases (MJD); the second group—the zinc metalloproteases—comprises the JAB1/MPN/Mov34 proteases, also known as JAMM [27]. Despite this classification, little is known about the physiological importance of many of them. Nevertheless, different processes such as endocytosis and trafficking of different receptors and modulation of signalling cascades have been shown to be regulated by deubiquitination. In this way, some receptor tyrosine kinases (e.g., the epidermal growth factor receptor, EGFR), internalisation is regulated via deubiquitinases such as USP9X, Cezanne-1 and -2, and USP2a, whereas Associated Molecule with the SH3 of STAM (AMSH) and USP8 regulate EGFR sorting [28]. Additionally, several studies suggest that the Nuclear Factor Kappa-light-chain-enhancer of activated B cells (NF-κB) pathway is also negatively modulated by deubiquitinases such as CYLD [29] and USP11 [30]. Other cascades that seem to be modulated by DUBs include the Wingless-related integration site (WNT) and the Transforming Growth Factor (TGFβ) signalling pathways (reviewed in [31]). Still, more research on this matter is required until all the physiological implications of DUBs are unveiled.

## 4. Neurotrophin Receptors Ubiquitination

### 4.1. p75^NTR^ Ubiquitination

Little is known about the p75^NTR^ ubiquitination and its modulation via Ub-binding. The first indication that p75^NTR^ could be modulated by ubiquitination was a report where the interaction of the E3 Ub-ligase c-Cbl with the pan-neurotrophin receptor was described [32]. The results obtained from PCNA and HEK293 cells in which p75^NTR^ was exogenously expressed showed that c-Cbl was able to bind to p75^NTR^, but only when the latter was phosphorylated on Ser-308. This phosphorylation—which was also found in p75^NTR^ from brain lysates—led to the receptor ubiquitination [32].

However, the very first interaction of an E3 Ub-ligase with p75^NTR^ was described previously [33], when the association with the tumour-necrosis factor receptor (TNFR)-associated factor-6 (TRAF6) was reported. In spite of this finding, no further analysis was carried out until 2004, when TRAF6 knock-out mice showed p75^NTR^-deficient signalling [34]. Subsequent studies have demonstrated that the modulation of p75^NTR^ by TRAF6 in HEK293 cells is through the polyubiquitination of K247, K280, and K283 [35]. Interestingly, this group also showed that this polyubiquitination induced the gamma-secretase-mediated proteolysis of p75^NTR^. The fact that TRAF6 is a modulator of the pan-neurotrophin receptor ubiquitination upon NGF stimulation has been reinforced by the experiments carried out by Geetha and collaborators. They suggest that the interaction of p75^NTR^ and TRAF6 may promote neuronal cell survival in HT-22 cells in an NGF-dependent manner [36]. In a parallel study, the same group analysed the effects of Amyloid-β on p75^NTR^ regulation. It is well established that Amyloid-β peptides aggregate and form the senile plaques in Alzheimer’s Disease (AD) [37]. Because it is also known that pro-NGF accumulates in AD [38,39], Geetha and collaborators assessed whether p75^NTR^ functions could be somehow affected upon the presence of amyloid-β in cells. Their experiments in HT-22 cells suggest that amyloid-β peptide may induce cell death through p75^NTR^, which could be counteracted by overexpression of TRAF6/p62 and subsequent receptor polyubiquitination and degradation [40]. Therefore, ubiquitination of p75^NTR^ may have a protective role in AD.

### 4.2. Ubiquitination of TrkA

To date, four different E3 Ub-ligases have been described that modulate the ubiquitination of the TrkA receptor, and thus its turnover. The first E3 Ub-ligase described for Trk receptors was TRAF6 (E3 RING-family). As previously mentioned, TRAF6 interacts with p75^NTR^, and this p75^NTR^/TRAF6 complex promotes K63-linked polyubiquitination, which seems to regulate the internalisation of TrkA, and afterwards, TrkA signalling [12]. These data suggesting that p75^NTR^ has a positive role on TrkA ubiquitination contradict another report [13]. In this latter study, the authors proposed that the pan-neurotrophin receptor interferes with TrkA and TrkB ubiquitination, impairing TrkA internalisation and protecting the receptor from degradation. In addition, the research of Geetha et al. presents another dilemma regarding the level of the TrkA receptor in mouse brain, as it has been reported that its expression is limited to specific areas as compared to TrkB or TrkC levels [41]. In Geetha’s study, an antibody was used to immunoprecipitate the TrkA receptor in brain lysates; however, its specificity comprises the recognition of TrkB and TrkC, and hence the interpretation of the results might be inaccurate. Finally, the adaptor protein p62 is required to form a complex with TRAF6 to permit the polyubiquitination event [12,42,43]. However, p62 alone seems to shuttle the polyubiquitinated TrkA towards the proteasome, which in turn promotes the lysosomal degradation of the receptor [44]. Taking this all into account, supplementary experiments are still required to fully understand the mechanism behind TRAF6/p62 involvement in the ubiquitination of Trks. Nevertheless, it appears that TRAF6 promotes K63-linked polyubiquitination in Trks, leading to both the regulation of receptors present on the cell membrane as well as NGF-mediated signalling, as seen in PC12 cells [12].

Furthermore, two forms of the E3 RING Ub-ligase Cbl, which participates in lysosomal degradation of tyrosine kinase receptors [45], have been described to be negative regulators of TrkA. These two forms are c-Cbl and Cbl-b, and have been shown to ubiquitinate TrkA upon NGF stimulation, inducing its subsequent lysosomal degradation and limiting NGF signalling within the cells [46,47]. Hence, the action of Cbl on TrkA limits NGF signalling within the cells.

Finally, Nedd4-2 is a member of the Nedd4 E3 HECT Ub-ligases family that has been shown to specifically ubiquitinate TrkA. Nedd4-2 binds to the PPXY TrkA motif (which is not present in TrkB or TrkC receptors), leading to the multi-monoubiquitination of TrkA [11]. Further support for the specificity of Nedd4-2 for TrkA was obtained by neuronal apoptosis induced in NGF-dependent dorsal root ganglion (DRG) neurons but not BDNF-dependent DRG neurons after overexpressing Nedd4-2 [11]. The involvement of Nedd4-2 in TrkA ubiquitination has again been supported by subsequent studies by other laboratories [47,48]. In addition, mutations surrounding the PPXY motif seem to increase Nedd4-2 binding to TrkA [48]. The effects of Nedd4-2 in TrkA-mediated functions have been assessed both in vitro and in vivo. Hence, the receptor trafficking, degradation, and NGF-mediated signalling are affected when the endogenous level of Nedd4-2 is depleted in TrkA-positive DRG neurons [49]. Using a mouse model that expresses a variant of the TrkA receptor containing a mutated copy of the binding motif for Nedd4-2 (TrkAP782S), which leads to its inefficient ubiquitination and degradation, reinforces the idea that Nedd4-2 influences TrkA in neuronal survival. Consequently, TrkA signalling is enhanced in vivo and results in an increased number of sensory neurons [50]. Moreover, the physiological relevance of TrkA ubiquitination has been demonstrated in the same mouse model, suggesting a higher sensitivity to thermal and inflammatory pain [50]. In addition, mutation of K450 in the mouse TrkA receptor resulted in reduced ubiquitination of the receptor that produced enhanced thermal sensitivity and inflammatory pain [51]. In rat TrkA, K447 (similar to K450 in mouse TrkA) and K775 have been shown to be ubiquitinated using mass spectrometry [46]. Taken together, these reports indicate that TrkA ubiquitination has profound effects on NGF-mediated functions in vivo and in vitro.

### 4.3. TrkB and TrkC Ubiquitination

So far, very little is known about the ubiquitination of TrkB in comparison to TrkA, and even less is known for TrkC. The first report describing TrkB ubiquitination following neurotrophin receptor activation appeared in 2005 [12,13]; this was later supported by the work of Arévalo and collaborators [11]. However, none of these reports identified an E3 Ub-ligase responsible for TrkB ubiquitination. Using bioinformatic approximations, the TRAF6/p62 complex was predicted as a putative E3 Ub-ligase for TrkB, and subsequent experiments confirmed the ubiquitination of the receptor at K811 in transfected HEK293 cells stimulated with BDNF [42]. In 2014, Pandya and collaborators carried out in vitro studies regarding TrkB ubiquitination. They found an interaction between TrkB and c-Cbl (E3 RING-family) and—after acute corticosteroid administration in cultured cortical neurons—increased TrkB levels were observed in tandem with an increase in c-Cbl levels and TrkB ubiquitination. By contrast, chronic treatment of cortical neurons in adult mice promoted a decrease in both TrkB and c-Cbl levels [52]. Thus, c-Cbl was identified as an E3 Ub-ligase for TrkB that was also regulated by glucocorticoids. This could be of interest in terms of the physiological response to an acute and chronic stress response and their implications in comorbidities, such as the development of depression after a long period of stress in which BDNF alterations are involved [53]. Recently, it has been observed that TrkB activation differs depending on whether BDNF or NT-4 binds to the receptor. This distinction seems to be dependent on varying degrees of TrkB ubiquitination, as a result of neurotrophin binding. While BDNF induces a very effective ubiquitination of the receptor and its downregulation, NT-4 causes slower ubiquitination and internalisation of TrkB receptor, and consequently a more sustained activation of downstream signalling [54]. The underlying mechanism behind this phenomenon remains unknown, despite both ligands having an equal affinity for binding TrkB.

Regarding TrkC ubiquitination, TRAF6/p62 complex has also been predicted to be a potential E3 Ub-ligase for TrkC (K602 and K815) using bioinformatic approximations and computational methods [42,43]; however, no experimental evidence has been provided. Laboratory analyses are needed to empirically confirm these predictions.

## 5. Deubiquitination of Trk Neurotrophin Receptors

As already mentioned, the Trk ubiquitination/deubiquitination cycle controls the fate of the receptor within the cell: internalisation, signalling, recycling, and degradation. The latter two events are carried out through different cycles of ubiquitination/deubiquitination. Firstly, the ubiquitinated receptor suffers an endocytic process where it is consequently removed from the cell surface. Then, removal of Ub from Trk receptors can occur via a DUB, and once it is removed it can be either recycled back to the membrane or degraded by the lysosome [55,56]. Hence, TrkA recycling and degradation in the lysosomes requires a previous deubiquitination process that directly depends on proteasome activity. Evidence for this has been provided by experiments where blockage of the proteasome increased TrkA recycling to the cytoplasmic membrane [57]. The question of which proteasome-related deubiquitinases participate in the lysosomal degradation of TrkA remains unanswered, although some deubiquitinases that modulate TrkA have already been identified.

The tumour suppressor gene CYLD (USP-family) was first described in familial cylindromatosis which causes skin neoplasia [58]. Afterwards, it was shown to play a role in modulating NF-κB signalling, promoting the deubiquitination of a modulator of this pathway (NEMO) [29]. Moreover, additional studies showed that CYLD specifically deubiquitinates polyubiquitin chains at K63 of different substrates (e.g., TRAF2 and TRAF6), or tyrosine kinase receptors such as TrkA. Geetha and collaborators saw that the polyubiquitination of TrkA increases when CYLD is depleted, but no direct evidence of TrkA deubiquitination by CYLD was provided [12]. This effect is not specific to the neurotrophin receptors, as downstream pathways from other tyrosine kinase receptors seem to be affected in a similar way when the K63-linked ubiquitin is removed [59]. The adaptor p62 protein appears to be necessary for CYLD K63 deubiquitinating function, as seen in the brains of p62 knock-out mice, where the poly-K63 ubiquitinated Trk receptors seem to accumulate [59]. Hence, more in-depth research is required concerning this subject.

Recent studies have attempted to identify the specific DUBs associated with TrkA, where Ceriani and collaborators described an interaction between TrkA and USP8 (USP-family) in PC12 cells [60]. Likewise, our group has performed a screen searching for putative DUBs that could regulate TrkA activation upon NGF stimulation [61]. In this study, several USPs were identified as strong candidates, of which the USP36 (USP-family) was studied in more detail. The results obtained provided convincing evidence that USP36 was capable of regulating TrkA. However, the effect produced by this DUB on the receptor was indirect, as it did not deubiquitinate TrkA. Instead, USP36 interfered with the binding of Nedd4-2 to TrkA, and hence USP36 expression impaired the Nedd4-2-mediated ubiquitination of TrkA [61]. Using mass spectrometry, it was determined that other DUBs seem to be recruited to TrkA in response to NGF [46]. However, no further studies have been carried out to address the significance of the interactions between TrkA and these DUBs. Since TrkA deubiquitination has been only studied in in vitro assays, further experiments are required to describe the in vivo physiological implications of these DUBs, as well as the identification of new DUBs specific for Trks.

## 6. Clinical Relevance and Future Approaches

Due to the broad spectrum of interrelated functions, disruption of just one enzyme related to ubiquitination can cause the development of abnormal cellular activities that can lead to an enormous number of disorders. For example, Parkin—an E3 Ub-ligase related to mitophagy—has been shown to be altered in Parkinson’s disease [62,63] and cancer [64]. Additionally, DUBs have been implicated in oncogenesis, so they are becoming potential therapeutic targets [65]. With respect to disorders affecting the central nervous system, current research is starting to shed light on this issue. For example, a direct relationship between Nedd4-2 and neuropathic pain has been described [66]. Neuropathic pain is produced after nerve injury with diverse aetiologies, being a common feature an abnormal sensory perception resulting in mechanical and thermal hyperalgesia and allodynia [67]. The causes related to this condition are not clear, although it has been suggested that an abnormal distribution of voltage-dependent sodium channels (Na_v_) due to an imbalance in Nedd4-2 Ub-ligase could play a role in nociception [66]. Nedd4-2 is implicated in the regulation of several different voltage-gated ion channels involved in neuronal excitability [68]. Therefore, it is probable that these channels are involved in neuropathic pain. This data proposes that the process of ubiquitination directly acts in pain modulation. Additionally, the experiments carried out with the TrkAP782S and TrkAΔKFG mice—which present altered TrkA ubiquitination—showed an increased sensitivity to pain upon heat and inflammatory stimuli [50,51]; these reports indicate an implication of TrkA ubiquitination in pain sensitivity. It is important to mention that the implication of Trk receptors in pain sensitivity, including neuropathic pain, has been already established [69]. For example, the inhibition of the NGF/TrkA pathway is one of the strategies currently under investigation to treat neuropathic pain [70]. Altogether, these findings are very interesting, as they may help to unravel the mechanisms of how chronic and neuropathic pain are developed and maintained, since approximately 20% of the population suffers from them. Taking this into account, it is not unreasonable that deubiquitinases like USP36 could also participate in nociceptive perception. Further experiments in both in vitro and in vivo models are required to fully understand the modulation of TrkA signalling via ubiquitination, and it is essential to characterise the Ub-ligases and DUBs that may be participating in TrkB and TrkC regulation. Finally, it would be interesting to assess the ubiquitin-related enzyme levels in human samples from patients with different nervous system disorders, such as Parkinson’s, Alzheimer’s, or psychiatric conditions.

## 7. Conclusions

Currently, the regulation of the neurotrophin receptors via ubiquitination remains unresolved, where the most studied receptor has been TrkA, for which four different E3 Ub-ligases have been described (TRAF6, Nedd4-2, c-Cbl, and Cbl-b). Furthermore, three DUBs (CYLD, USP8, and USP36) whose mechanisms might be specific and non-specific for the NGF receptor (Table 1 and Table 2) have been reported. However, very little is known about the TrkB and TrkC receptors, and the ubiquitination system of p75^NTR^ is still doubtful. Considering the strong evidence of the role of Nedd4-2 in modulating nociception, a logical question would be whether there is a DUB that could also be implicated in the process. Since there is currently no effective treatment against neuropathic pain and other forms of chronic pain [71], Ub-ligases and DUBs should be considered as potential therapeutic targets. Similarly, it is necessary to continue studying this posttranslational modulatory system for which the clinical relevance seems to be rather clear. 

## Figures and Tables

**Table 1:** Summary of the E3 Ub-ligases that participate in the ubiquitination of neurotrophin receptors.

Neurotrophin Receptor	Ub-Ligase	Ub-Ligase Family	References
p75^NTR^	c-Cbl	RING-family	[32]
	TRAF6	RING-family	[33,34,35,36]
TrkA	TRAF6	RING-family	[12,13,42,43]
	c-Cbl	RING-family	[47]
	Cbl-b	RING-family	[46]
	Nedd4-2	HECT-family	[11,48,49,50]
TrkB	TRAF6	RING-family	[42,43]
	c-Cbl	RING-family	[52]
TrkC	c-Cbl *	RING-family	[52]
	TRAF6 *	RING-family	[42,43]

* The identification of Ub-ligases for TrkC have been only predicted through bioinformatic tools. RING: really interesting new gene.

**Table 2:** Deubiquitinating enzymes that participate in the removal of ubiquitin on the TrkA receptor.

Neurotrophin Receptor	Deubiquitinase	DUB Family	References
TrkA	CYLD	USP-family	[12,59]
	USP8	USP-family	[60,61]
	USP36 *	USP-family	[61]

* Notice that the effect of USP36 on TrkA is indirect, as its mechanism of action is based on the impairment of the Nedd4-2/TrkA interaction.
